# Impact of Chemical Disinfectants on Surface Microhardness of Acrylic Resin Denture Teeth: An In Vitro Study

**DOI:** 10.7759/cureus.88801

**Published:** 2025-07-26

**Authors:** Anche Sampath, Prathista Velaga, Samyuktha Devarapalli, Sadvi Guntupalli, Rahul B Peeta, Hita Boyapati

**Affiliations:** 1 Department of Prosthodontics, Sibar Institute of Dental Sciences, Guntur, IND

**Keywords:** acrylic resin, chemical, denture base, disinfectant, hardness

## Abstract

Introduction: This in vitro study aimed to evaluate the effects of three chemical disinfectants, glutaraldehyde, sodium hypochlorite, and chlorhexidine gluconate, on the surface microhardness of acrylic resin denture teeth. The objectives of this study were to assess and compare the impact of these disinfectants on microhardness following repeated disinfection cycles.

Materials and methods: This in vitro study was conducted at the Department of Prosthodontics, involving 76 acrylic resin denture teeth (Premadent, Premadent Dental Products, Delhi, India) embedded in self-cure acrylic resin (DPI-RR Cold Cure, Dental Products of India, Mumbai, India) using a vinyl polysiloxane putty mold (Elite HD+ Putty, Zhermack SpA, Badia Polesine, Italy). The samples were divided into four groups (n = 19 each): Group 1 (control) was immersed in distilled water (PureFlow Distilled Water, Aquaguard, Mumbai, India); Group 2 was immersed in 2% glutaraldehyde (Cidex, Johnson & Johnson, New Brunswick, NJ, USA); Group 3 was immersed in 1% sodium hypochlorite (Clorox, The Clorox Company, Oakland, CA, USA); and Group 4 was immersed in 2% chlorhexidine gluconate (Hexidine, ICPA Health Products Ltd., Mumbai, India). Each group underwent three 10-minute disinfection cycles at a seven-day interval and was stored in distilled water between cycles. Surface microhardness was measured using a Vickers hardness tester (HMV-2T, Shimadzu Corporation, Kyoto, Japan) after the first and third cycles. Data were analyzed using one-way analysis of variance (ANOVA), Tukey’s post-hoc test, and paired t-tests (IBM SPSS Statistics for Windows, Version 23 (Released 2015; IBM Corp., Armonk, New York, United States).

Results: The control group showed a significant reduction in microhardness from the first cycle to the third cycle (p = 0.00, large effect size). The glutaraldehyde and sodium hypochlorite groups also exhibited significant decreases (p < 0.05, large effect size). No significant changes were observed in the chlorhexidine gluconate group (p = 0.328; d = 0.23). Significant intergroup differences were observed in the first cycle (p = 0.001), but not in the third cycle (p = 0.145), indicating a convergence of effects over time.

Conclusion: Glutaraldehyde and sodium hypochlorite significantly reduced the microhardness of acrylic resin denture teeth, whereas chlorhexidine gluconate had a minimal effect, suggesting its suitability for routine denture disinfection. Careful selection of disinfectants is crucial for maintaining the integrity of denture materials.

## Introduction

Acrylic resin denture teeth are widely used in prosthodontics due to their favorable properties, including cost-effectiveness, ease of fabrication, and aesthetic appeal [1]. These materials are designed to mimic natural teeth in function and appearance, making them a popular choice for removable and fixed dental prostheses [1]. However, the long-term performance of acrylic resin denture teeth depends on their ability to withstand the oral environment, including exposure to chemical disinfectants used for denture hygiene [2]. Maintaining proper denture hygiene is critical to prevent microbial colonization, which can lead to oral infections such as denture stomatitis, a common condition affecting denture wearers [3]. Chemical disinfectants, such as glutaraldehyde, sodium hypochlorite, and chlorhexidine gluconate, are routinely employed to ensure microbial control [4]. However, these solutions may inadvertently affect the physical and mechanical properties of acrylic resin denture teeth, including surface microhardness, a key indicator of material durability and wear resistance [5].

Surface microhardness reflects a material’s ability to resist indentation and scratching, directly influencing its longevity and clinical performance [6]. Prolonged or improper disinfectant use may degrade surfaces, reducing microhardness, increasing roughness, and promoting microbial adhesion. Glutaraldehyde, a high-level disinfectant, is known for its broad-spectrum antimicrobial activity but may cause chemical interactions with polymer structures [4]. Sodium hypochlorite, a commonly used bleaching and disinfecting agent, is effective against bacteria and fungi but has been associated with surface deterioration of acrylic resins due to its oxidative properties [5,6]. In contrast, a previous study has reported no significant color change of denture base resins with 0.25% sodium hypochlorite [7]. The interaction between these disinfectants and acrylic resin denture teeth is complex, and their impact on surface microhardness remains an area of active investigation.

Several studies have explored the effects of disinfectants on acrylic resin denture materials, focusing on properties such as color stability, surface roughness, and flexural strength [5-7]. For instance, Davi et al. [5] and Paranhos et al. [6] reported that sodium hypochlorite caused significant surface alterations in acrylic resins. The degree of change varied depending on the concentration of sodium hypochlorite and the duration of immersion [6]. However, the specific effects of these disinfectants on the surface microhardness of acrylic resin denture teeth have received limited attention. Most studies have focused on denture base materials rather than denture teeth, which differ in composition and processing, limiting the applicability of findings to teeth. Furthermore, comparative analyses of glutaraldehyde, sodium hypochlorite, and chlorhexidine gluconate in a single study are scarce, leaving a gap in understanding their relative impacts on microhardness. This gap in comprehensive data limits evidence-based recommendations for disinfectant use in denture hygiene protocols. This in vitro study addressed this research gap by evaluating and comparing the effects of three commonly used chemical disinfectants, such as glutaraldehyde, sodium hypochlorite, and chlorhexidine gluconate, on the surface microhardness of acrylic resin denture teeth. 

## Materials and methods

Study design and setting

This in vitro experimental study was conducted at the Department of Prosthodontics, Sibar Institute of Dental Sciences, Guntur, India. Ethical clearance was not required for this in vitro study because it did not involve human or animal subjects and adhered to the institutional guidelines for the research. The study duration was five months from January 2024 to May 2024, encompassing sample preparation, disinfection cycles, and microhardness testing. The design followed a controlled experimental approach, with the samples divided into four groups to assess the impact of disinfectants compared to the control group immersed in distilled water.

Sample size calculation

Sample size was calculated using G*Power software version 3.6.9 (Heinrich-Heine-Universität Düsseldorf, Düsseldorf, Germany). The sample size was calculated using an a priori analysis of variance (ANOVA) fixed-effect omnibus design based on a previous study that evaluated the effect of disinfectants on acrylic resin denture teeth with an effect size of 0.40 [8]. Using a power of 80% and an alpha error of 0.05, a minimum of 15 samples per group were determined to detect a clinically significant difference in Vickers hardness number (VHN). To account for potential sample loss or experimental errors, 19 samples were included per group, resulting in 76 samples.

Methodology

A wax block measuring 20 mm × 20 mm × 6 mm was fabricated using modelling wax (Y-Dents Modelling Wax, MDM Corporation, Delhi, India). The wax block was indexed using a vinyl polysiloxane putty material (Elite HD+ Putty, Zhermack SpA, Badia Polesine, Italy) to create a mold with consistent sample dimensions. The putty index was prepared by impressing a wax block onto the putty material, ensuring uniform mold cavities for subsequent acrylic resin pouring. The mold was inspected for accuracy and cleaned to remove any residual wax particles.

Acrylic resin denture teeth (Premadent, Premadent Dental Products, Delhi, India) consisting of mandibular central and lateral incisors were selected because of their relatively flat labial surfaces, which facilitated microhardness measurements without additional grinding or polishing. Self-cure acrylic resin (DPI-RR Cold Cure, Dental Products of India, Mumbai, India) was mixed according to the manufacturer’s instructions (polymer-to-monomer ratio of 3:1 by volume) and poured into the putty index. The acrylic resin denture teeth were embedded in a self-curing resin with the labial surface facing upward. After curing at room temperature for 15 min, specimens were removed from the putty index. Uneven or sharp edges were trimmed using an acrylic trimmer (Marathon-3; Saeyang Microtech, Seoul, South Korea) and polished with 600-grit silicon carbide paper (3M India Ltd., Bangalore, India) to ensure a smooth and standardized surface.

All 76 samples were initially stored in distilled water (PureFlow Distilled Water; Aquaguard, Mumbai, India) at room temperature (25 ± 2 ^0^C) for 24 h to simulate the baseline hydration. The samples were then divided into four groups (n = 19 each) and subjected to disinfection cycles as follows: Group 1 (Control), immersed in distilled water (PureFlow Distilled Water, Aquaguard, Mumbai, India); Group 2 was immersed in 2% glutaraldehyde solution (Cidex, Johnson & Johnson, New Brunswick, NJ, USA); Group 3 was immersed in 1% sodium hypochlorite solution (Clorox, The Clorox Company, Oakland, CA, USA); and Group 4 was immersed in 2% chlorhexidine gluconate solution (Hexidine, ICPA Health Products Ltd., Mumbai, India).

Each group was immersed in the respective solution for 10 min at room temperature. A 10-minute immersion time was selected to standardize exposure across all groups and ensure valid comparison. After immersion, the samples were washed under running tap water for 30 s, dried with compressed air jets, and stored in distilled water at room temperature for seven days. This process consisted of one disinfection cycle. Three cycles were performed (C1 and C3 for the control; G1, G3 for glutaraldehyde; S1, S3 for sodium hypochlorite, and H1 and H3 for chlorhexidine) with a seven-day interval between each cycle. After the third cycle, the samples were stored in distilled water for an additional seven days before the final test. Three disinfection cycles with seven-day intervals were designed to simulate repeated clinical disinfection over time, reflecting real-life denture hygiene practices. The seven-day interval allowed for material rehydration and stabilization between cycles, reducing short-term transient effects and better mimicking routine weekly cleaning. An additional seven-day storage in distilled water after the final cycle ensured equilibration and eliminated any immediate post-disinfection surface changes, allowing for standardized and reliable final microhardness measurements.

**Figure 1:**
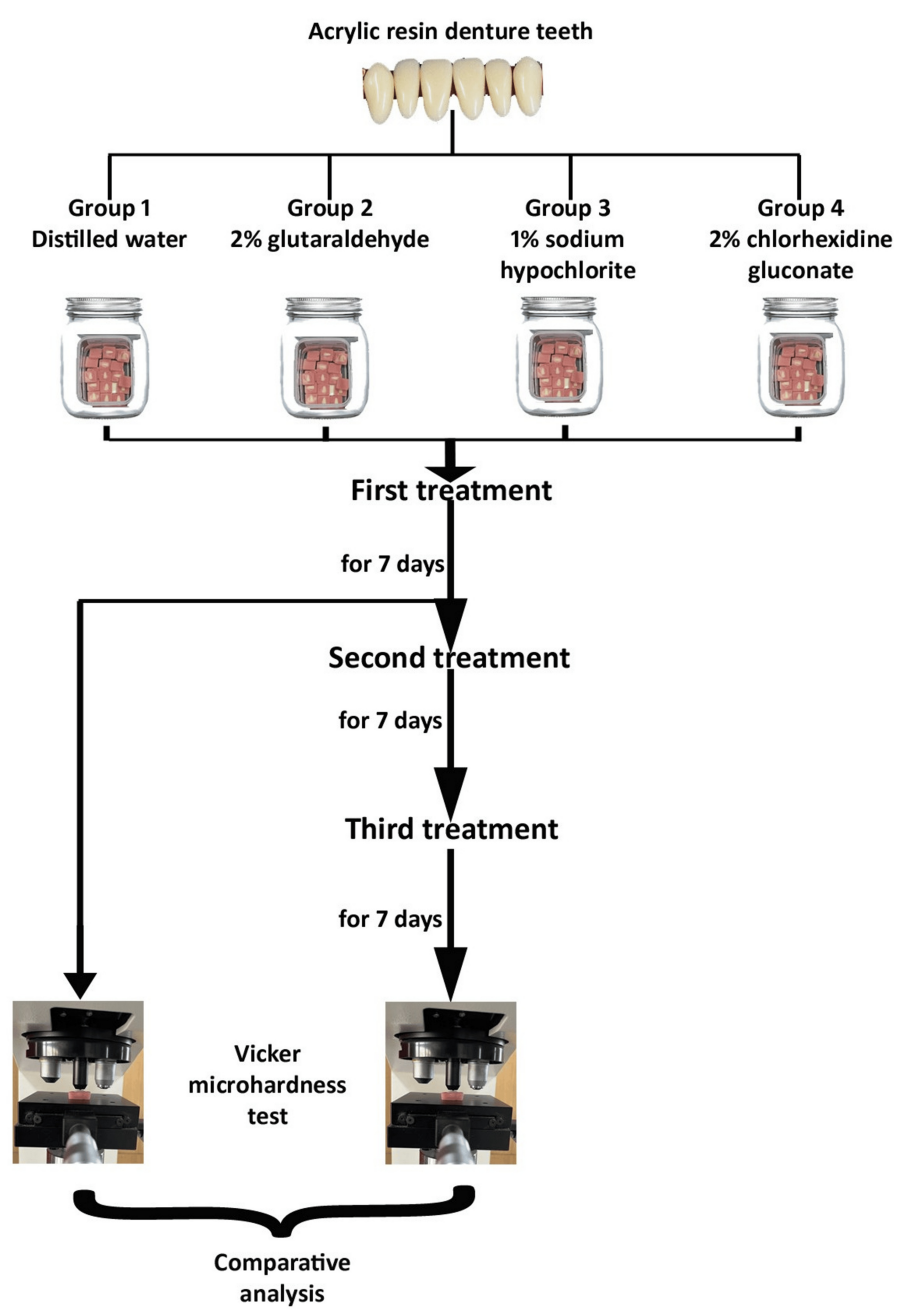
Flowchart of the study protocol. Original image created by authors.

Surface microhardness was measured using a Vickers hardness tester (HMV-2T, Shimadzu Corporation, Kyoto, Japan) equipped with a diamond indenter. Measurements were performed after the first cycle (C1, G1, S1, and H1) and after the third cycle (C3, G3, S3, and H3). A load of 50 g (0.5 N) was applied for 10 seconds on the labial surface of each tooth. Three indentations were made at distinct locations (the cervical, middle, and incisal edges) on the labial surface to account for potential surface variations. The indentations were observed using the integrated optical microscope attached to the Vickers hardness tester, equipped with a high-precision measurement system and 10x objective lens, allowing accurate visualization and measurement of indentation diagonals with a resolution of up to 0.1 µm. The arithmetic mean of the three measurements was calculated to determine the VHN for each sample (Figure 1).

Reliability and calibration

To ensure reliability, a single trained examiner performed all microhardness measurements to minimize inter-examiner variability. The Vickers hardness tester was calibrated before each testing session by using a standard reference block provided by the manufacturer (Shimadzu Corporation, Kyoto, Japan). The calibration process involved verifying the accuracy of the indenter and load application, ensuring that the measurements were within ±2% of the reference standard. The examiner underwent training to standardize the indentation technique and record measurements. A pilot test on five additional acrylic resin denture teeth was conducted to confirm intra-examiner reliability, achieving an intraclass correlation coefficient of 0.92, indicating high reproducibility.

Statistical analysis

Data were analyzed using IBM SPSS Statistics for Windows, Version 23 (Released 2015; IBM Corp., Armonk, New York, United States). The mean VHN in each group was calculated after the first and third cycles. Continuous microhardness data were subjected to the Shapiro-Wilk test to assess normal distribution, confirming that the data followed a normal pattern, thus justifying the use of parametric tests. Intergroup comparisons were performed using one-way ANOVA supplemented by post hoc analysis using Tukey’s test to identify specific differences. Intragroup comparisons were performed using paired t-tests to examine within-group differences. A significance level of p < 0.05 was established for all statistical tests.

## Results

A comparison of microhardness between the first and third treatments across the different study groups is presented in Table 1. The control group showed a significant reduction in microhardness from the first to the third treatments (p = 0.00; large effect size). Similarly, the glutaraldehyde and sodium hypochlorite groups exhibited a significant decrease from the first to the third treatment (p < 0.05), with large effect sizes. In contrast, no significant change in microhardness was observed in the chlorhexidine gluconate group (p = 0.328, d = 0.23), suggesting a negligible effect. These findings indicated that repeated treatments with control, glutaraldehyde, and sodium hypochlorite significantly reduced dentin microhardness, whereas the chlorhexidine gluconate group showed stability, possibly because of its milder action on acrylic resin.

**Table 1:** Comparison of microhardness in the Vickers hardness number (VHN) between first and third treatments for different study groups. *p-value < .05 denotes statistical significance using a paired t-test. Data are presented as mean and standard deviation (SD), where n indicates the number of samples in each group.

Groups	Time of treatment	n	Mean	SD	t value	p-value	Cohen d
Control (Distilled water)	First treatment	19	25.80	2.21	3.49	0.003*	0.80
	Third treatment	19	22.91	3.61			
2% Glutaraldehyde	First treatment	19	27.01	2.62	3.48	0.003*	0.80
	Third treatment	19	23.72	4.23			
1% Sodium hypochlorite	First treatment	19	23.11	1.94	3.26	0.004*	0.75
	Third treatment	19	21.41	2.38			
2% Chlorhexidine gluconate	First treatment	19	22.67	2.04	1.01	0.328	0.23
	Third treatment	19	22.23	1.77			

In the first treatment, analysis revealed a significant difference between the groups (p = 0.001). In contrast, in the third treatment, the difference was not significant (p = 0.145), suggesting uniformity across the groups over time. These results implied that the initial treatment effects on microhardness varied significantly between groups, potentially due to differing compositions of treatment groups, while the lack of significance at the third treatment might reflect a stabilization or convergence of effects (Table 2).

**Table 2:** The intergroup (between study groups) comparison of microhardness at two different treatment incidences. *p-value < .05 denotes statistical significance using one-way analysis of variance (ANOVA), df denotes difference.

Treatment	Sum of Squares	df	Mean Square	F value	p-value
First treatment	250.43	3	83.48	16.92	0.001*
Third treatment	55.24	3	18.41	1.86	0.145

The post hoc Tukey’s test for microhardness at the first treatment incidence, as shown in Table 3, revealed significant differences among the study groups. Comparison of the control vs. sodium hypochlorite and control vs. chlorhexidine gluconate groups showed a statistically significant difference (p < 0.05). Similarly, glutaraldehyde vs. sodium hypochlorite and glutaraldehyde vs. chlorhexidine gluconate showed notable differences. However, the control vs. glutaraldehyde (p = 0.593) and sodium hypochlorite vs. chlorhexidine gluconate (p = 1) groups showed no significant differences. These findings suggested that the effects of the initial treatment on microhardness varied significantly between certain groups, particularly those involving chlorhexidine gluconate (Table 3).

**Table 3:** Pairwise comparison using post hoc Tukey’s test for the first treatment incidence. *p-value < .05 denotes statistical significance using Tukey's test, CI denotes confidence interval.

Pairwise	Mean difference	t value	p-value	95% CI lower limit	95% CI upper limit
Control (Distilled water) vs. 2% Glutaraldehyde	1.20	-1.67	0.593	-3.20	0.79
Control (Distilled water) vs. 1% Sodium hypochlorite	2.69	3.74	0.002*	0.70	4.69
Control (Distilled water) vs. 2% Chlorhexidine gluconate	3.13	4.35	0.001*	1.14	5.12
2% Glutaraldehyde vs. 1% Sodium hypochlorite	3.90	5.41	0.001*	1.91	5.89
2% Glutaraldehyde vs. 2% Chlorhexidine gluconate	4.34	6.02	0.001*	2.34	6.33
1% Sodium hypochlorite vs. 2% Chlorhexidine gluconate	0.44	0.61	1.000	-1.55	2.43

## Discussion

The findings of the present study revealed significant variations in microhardness changes across the disinfectant groups, particularly highlighting the minimal impact of chlorhexidine gluconate compared to the more pronounced effects of glutaraldehyde and sodium hypochlorite. These results contribute to the understanding of disinfectant interactions with acrylic resin materials commonly used in prosthodontics and offer insights into their clinical implications for denture maintenance.

Acrylic resin dentures are widely used owing to their cost-effectiveness, ease of fabrication, and aesthetic properties [1]. However, their susceptibility to surface degradation under chemical exposure, such as with disinfectants, raises concerns about their long-term durability and functionality [2]. Surface microhardness is a critical parameter because it reflects the resistance of the material to wear, scratching, and deformation, which are essential for maintaining occlusal integrity and aesthetic longevity. The significant reduction in microhardness observed in the control, glutaraldehyde, and sodium hypochlorite groups after repeated disinfection cycles suggested that these solutions might compromise the surface integrity of the acrylic resin over time [4-6]. The reduction in microhardness seen in vitro may reflect clinical outcomes, as dentures are repeatedly exposed to disinfectants. Over time, this could lead to surface degradation, increased wear, and reduced longevity of acrylic denture teeth in actual use. In contrast, the stability of the microhardness in the chlorhexidine group indicated a potentially safer profile for routine denture disinfection.

The control group immersed in distilled water exhibited a significant decrease in microhardness, which may seem counterintuitive because water is generally considered inert. However, studies have suggested that prolonged water immersion can lead to water sorption in acrylic resins, causing plasticization and weakening of the polymer matrix [9]. This phenomenon may explain the observed reduction as water molecules penetrate the resin, reducing the intermolecular forces and surface hardness. The behavior of the control group underscores the importance of considering baseline material changes in experimental designs, as even neutral storage conditions can influence the outcomes.

Glutaraldehyde, a high-level disinfectant commonly used in medical and dental settings, significantly reduced the microhardness in this study. This aligns with previous research, indicating that glutaraldehyde can interact with the organic components of acrylic resins, leading to surface softening [10]. The aldehyde groups in glutaraldehyde may react with the polymer chains, causing cross-linking or chain scission, which compromises the mechanical properties of the material. A previous study reported that chemically polished heat-cured acrylic resins did not release glutaraldehyde after different periods of immersion compared with mechanical polishing [11]. This discrepancy may be attributed to differences in immersion duration, concentration, or resin composition, highlighting the need for standardized protocols in disinfectant studies.

Sodium hypochlorite, another widely used disinfectant, also significantly decreased microhardness, which is consistent with its aggressive chemical nature. The oxidative properties of sodium hypochlorite can degrade the polymer matrix of acrylic resins, leading to surface erosion and reduced hardness [12]. Hypochlorite ions may break ester bonds in the resin, causing microcracks and surface deterioration. Supporting this, a study by Davi et al. [5] found that sodium hypochlorite immersion led to significant surface alterations in acrylic denture bases, including increased roughness and reduced mechanical strength. However, a previous study reported that 0.2% sodium hypochlorite did not produce adverse effects on acrylic resins, indicating that the impact of sodium hypochlorite is dose- and time-dependent [13]. The present study’s use of a 1% concentration and 10-minute immersion cycles may have amplified its degradative effects, emphasizing the need for cautious application in clinical practice.

In contrast, chlorhexidine gluconate showed no significant change in microhardness, suggesting a milder interaction with acrylic resin. This finding is supported by studies demonstrating the compatibility of chlorhexidine gluconate with dental polymers, attributing its minimal impact to its cationic nature, which limits penetration into the hydrophobic resin matrix and provides better color stability [14,15]. The antimicrobial efficacy of chlorhexidine gluconate, combined with its apparent safety for acrylic surfaces, makes it a promising option for denture disinfection. A study by Fotovat et al. [16] reported increased color alteration with 2% chlorhexidine gluconate while exhibiting the least impact on surface roughness. These variations may stem from differences in chlorhexidine concentration, immersion frequency, or the presence of additives in commercial formulations, warranting further investigation.

The initial significant differences in microhardness among the groups in the first cycle, which diminished by the third cycle, suggest a convergence of the effects over time. This could indicate that the impact of disinfectants on surface properties reaches a plateau, possibly owing to the saturation of chemical interactions or stabilization of the surface layer of the resin. Alternatively, this may reflect cumulative degradation in the control group, aligning its microhardness with that of the disinfectant groups. This observation aligns with that of Pavarina et al. [17], who noted that repeated disinfection cycles led to increased surface changes across different solutions, suggesting a continuous decrease in hardness with aging.

The clinical implications of these findings are significant. Reduced microhardness can lead to increased wear, surface roughness, and microbial adhesion, thereby compromising the longevity and hygiene of dentures. Reduced microhardness makes denture surfaces more prone to wear and roughness, which can promote microbial adhesion. This increases the risk of infections like denture stomatitis and compromises the denture’s hygiene, durability, and overall clinical performance. Sodium hypochlorite and glutaraldehyde, while effective disinfectants, may require careful consideration of their concentrations and exposure times to minimize damage. Chlorhexidine gluconate, due to its minimal impact on microhardness, may be a more suitable choice for routine denture care, especially for patients who require frequent disinfection. However, clinicians must balance antimicrobial efficacy with material compatibility because the antifungal activity of chlorhexidine gluconate is less potent than that of sodium hypochlorite [18]. Surface roughness and hardness are key factors in the performance of acrylic denture teeth. Increased roughness promotes plaque buildup and microbial adhesion, raising the risk of infections. Reduced hardness leads to faster wear and surface deterioration, affecting function and longevity. Together, they impact denture hygiene, durability, and patient comfort.

The limitations of this study include its in vitro design, which might not fully replicate oral conditions, such as saliva, temperature fluctuations, or mechanical stresses. Additionally, the study focused solely on microhardness, omitting other properties such as surface roughness or color stability, which are also critical for denture performance. Future studies should explore these parameters and investigate their long-term effects under clinical conditions. Comparative studies with newer disinfectants such as quaternary ammonium compounds or enzymatic solutions could further inform optimal denture-care protocols.

## Conclusions

In conclusion, this study demonstrated that glutaraldehyde and sodium hypochlorite significantly reduced the surface microhardness of acrylic resin denture teeth, whereas chlorhexidine gluconate exhibited a minimal impact, suggesting its suitability for routine denture disinfection. These findings highlight the importance of selecting disinfectants that balance antimicrobial efficacy with material compatibility to ensure longevity and functionality of acrylic resin dentures. Clinicians should consider the potential for surface degradation when recommending disinfection protocols that favor solutions, such as chlorhexidine gluconate, for regular use. Further research is warranted to explore the long-term effects under clinical conditions and evaluate additional disinfectants to optimize denture care practices.
